# Chinese herbal medicine for gout: a review of the clinical evidence and pharmacological mechanisms

**DOI:** 10.1186/s13020-020-0297-y

**Published:** 2020-02-12

**Authors:** Xiansu Chi, Hongxiu Zhang, Shuo Zhang, Ke Ma

**Affiliations:** 1 Shandong Co-Innovation Center of Classic TCM Formula, Shandong University of Traditional Chinese Medicine, No. 4655, University Road, Changqing District, Jinan, 250355 Shandong China; 2 Institute of Virology, Jinan Municipal Center for Disease Control and Prevention, Jinan, 250021 People’s Republic of China

**Keywords:** Gout, *Bi* pattern, Traditional Chinese medicine, Pharmacological mechanism

## Abstract

Gout is a common arthritis condition due to disorders of purine metabolism and decreased uric acid excretion. Although researchers have carried out various studies on this disease, there are no effective drugs for patients with gout. In traditional Chinese medicine (TCM), gout pertains the category of *Bi* pattern due to qi stagnation in the meridians and collaterals. Chinese herbal medicinals has been employed to treat *Bi* patterns since the ancient China. In recent decades, classical TCM formulas and agents isolated from some Chinese herbal medicinals have been applied to treat gout and have achieved satisfactory effect. In this review, we focus on recent studies of gout in which TCM formulas were applied to treat animal models or to treat patients, and summarize the mechanism of gout from TCM perspective, the clinical application, pharmacological mechanism and the chemical compounds of TCM formulas in treating gout. In conclusion, through this study, we summarized the application principle of TCM formulas in gout treatment and some key issues of current research, and we hope this study will provide some references for applying TCM formulas to treat gout and will lay a foundation for the development of novel formulas for gout treatments.

## Background

Gout is a common arthritis condition due to deposition of monosodium urate (MSU), and is closely related to hyperuricemia [1]. Because of disorders of purine metabolism or impaired renal excretion [2, 3], serum uric acid levels rise to be abnormally high, and finally there would be the deposition of uric acid and acute persistent inflammatory reactions in the joints [4]. The most common clinical manifestations of gout are acute burning sensation and swelling of the joints. In some cases, clinical manifestations such as joint damage and renal dysfunction are often present [5]. Gout affects a significant portion of the population worldwide annually [6], and according to the estimates of World Health Organization (WHO), 3.9% of people worldwide are suffering from gout [7]. The acute onset of arthritis, joint malformations, chronic joint injuries and the formation of renal calculus not only reduce the quality of life, but also lead to disability [8].

Currently, although researchers have carried out various studies on this disease, there are no effective drugs for patients with gout. Drugs like colchicines, corticosteroids and non-steroidal anti-inflammatory drugs (NSAIDs) have been applied to treat acute onset of gout [9]. These drugs show good effect in the short term, but when used for a long time, gastrointestinal reactions, rashes, systemic vacuities and even renal failure would occur [10, 11]. Besides, such drugs could not prevent, halt or reverse the progression of this complicated disease. Therefore, therapies or drugs which can intervene the development of gout are greatly needed in clinic.

**Fig. 1:**
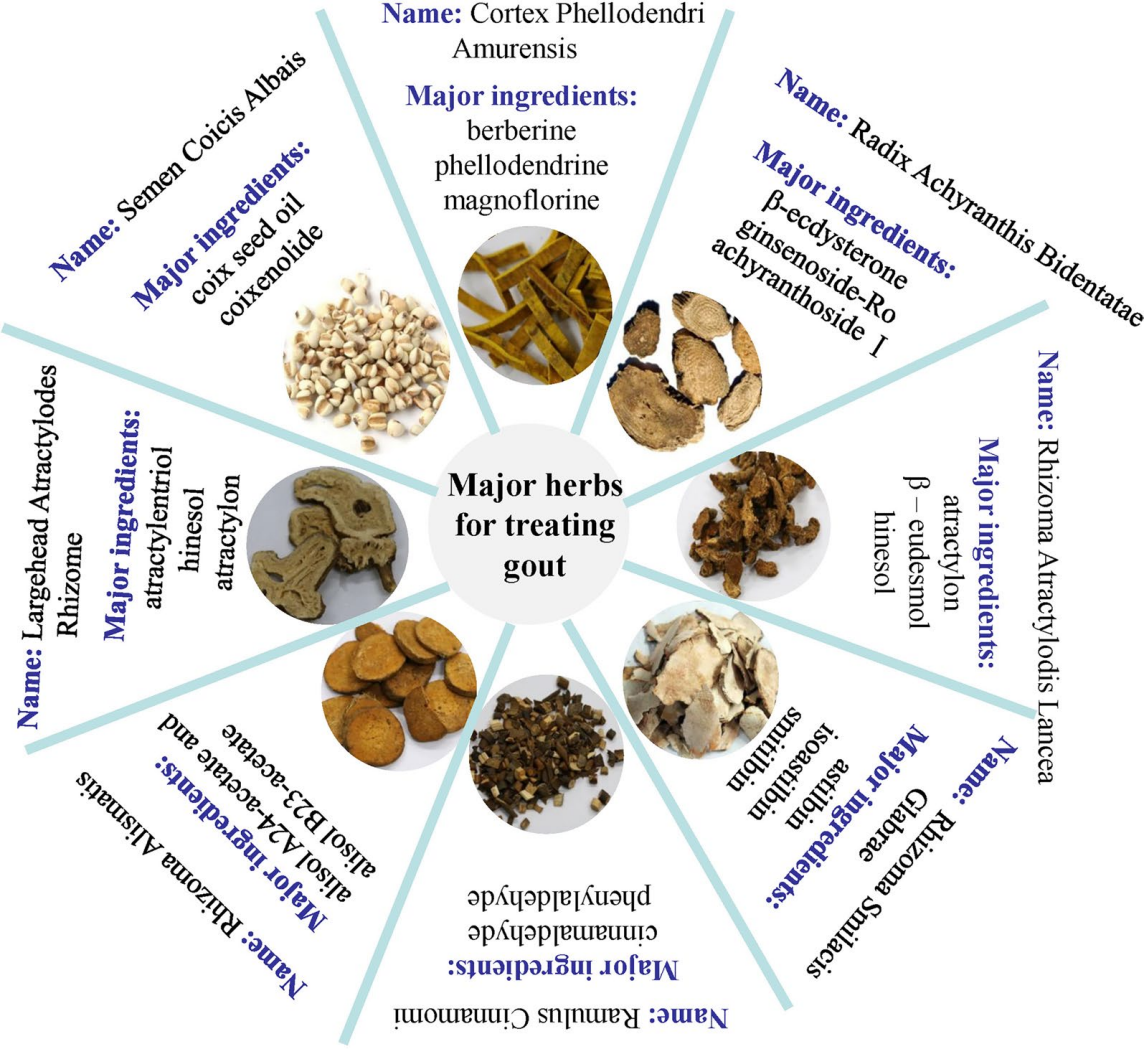
The commonly used ingredients isolated from TCM formulas or Chinese herbal medicinals for the treatment of Gout. Eight herbs have been frequently used for gout treatment with low toxicity and excellent effect under the principle of syndrome differentiation and treatment

Traditional Chinese medicine (TCM) has been applied to treat gout since the ancient China [12]. In TCM, gout is classified as *Bi* pattern usually due to qi stagnation in the meridians and collaterals. Under the guidance of pattern differentiation, classical TCM formulas and agents isolated from some herbs have been applied to treat gout with satisfactory effect [5]. Some chemical ingredients isolated from these formulas or Chinese herbal medicinals are of multi-target and low toxicity, showing advantages and good prospects in the prevention and treatment of gout (Fig. 1). In this review, the clinical application and pharmacological mechanism of TCM formulas in the treatment of gout are summarized, so as to develop novel formulas for gout treatments (Table 1).

**Table 1:** Ingredients of commonly used TCM formulas for the treatment of Gout

Formulas	Components	TCM efficacy	Pharmacological mechanism
*Jiawei Simiao* Powder together with external application of *Jinhuang* Cream [18]	*Jiawei Simiao* Powder: *Cortex Phellodendri Amurensis*, Semen Coicis, *Radix Achyranthes Root*, *Rhizoma Atractylodes Lanceae* and the composition of the formula should be adjusted according to clinical manifestations Jinhuang Cream: *Radix Angelicae Formosanae*, *Cortex Phellodendri Amurensis*, *Radix et Rhizoma Rhei Palmati*, *Rhizoma Curcumae Longae*, *Rhizoma Atractylodes Lanceae*, *Pericarpium Citri Reticulatae*, *Fied Rhizoma Arisaematis*,*Radix Trichosanthis*, *Cortex Magnoliae Officinalis*, *Radix et Rhizoma Glycyrrhizae*	*Jiawei Simiao* Powder: clearing away heat and draining dampness, promoting blood circulation and relieving swelling and pain *Jinhuang* Cream: clearing away heat and detoxifying, relieving swelling and pain, and dredging the collaterals to stop pain	Reducing the levels of CRP, ESR, IL-8 and SUA and relieving body inflammation
*Tongfeng* Decoction [19]	*Cortex Phellodendri Amurensis*, *Pseudobullbus Cremastrae*, *Radix Scrophulariae*, *Semen Coicis*, *Sclerotium Poriae Cocos*, *Rhizoma Alismatis*, *Herba Plantaginis*, *Caulis Lonicerae*, *Radix Stephaniae Tetrandrae*, *Radix Paeoniae Rubra*, *Radix Rehmanniae*, *Cortex moutan Radicis*, *Radix Angelica sinensis*, *Radix Achyranthes root*, *Radix et Rhizoma Glycyrrhizae Praeparata cum Melle*	Clearing away heat and draining dampness, eliminating turbid and dredging the collaterals to stop pain	Effectively reducing the acute inflammation index in the patient’s blood, inhibiting the body’s inflammatory response, inhibiting the progression of the disease, and reducing uric acid level
*Danxi Tongfeng* Decoction [20]	*Arisaema With Bile*, *Rhizoma* Atractylodes *Lanceae*, *Cortex Phellodendri Amurensis*, *Massa Medicata Fementata*, *Rhizoma Chuanxiong*, *Radix Stephaniae Tetrandrae*, *Semen Persicae*, *Radix Angelicae Formosanae*, *Ramulus Cinnamomi*, *Radix et Rhizoma Clematidis Chinensis*, *Rhizoma et Radix Notopterygii*, *Flos Carthami*, *Radix et Rhizoma Gentianae*	Invigorating spleen to eliminate dampness, dispelling wind and draining dampness, clearing away heat and detoxifying, and dredging the collaterals to stop pain	Reducing ESR and promoting uric acid excretion, and strong analgesic effect
*Wuwei Xiaodu* Decoction [51]	*Flos Lonicerae*, *Flos Dendranthematis Indici*, *Herba Taraxaci Mongolici*, *Herba Violae Philippicae*, *Radix Semiaquilegiae*	Clearing away heat and detoxifying	Reducing the IL-10 level to be normal, increasing phagocytosis index and phagocytosis rate of macrophages, thereby enhancing immune function
Modified *Wuwei Xiaodu* Decoction [21]	*Flos Lonicerae*, *Rhizoma Alismatis*, *Rhizoma Dioscoreae*, *Cortex Phellodendri Amurensis*, *Sclerotium Polypori Umbellati*, *Rhizoma Atratylodis Macrocephalae*, *Sclerotium Poriae Cocos*, *Herba Plantaginis*, *Herba Taraxaci Mongolici*, *Herba Violae Philippicae*, *Nudicaulous Grounsel Herb*, *Flos Dendranthematis Indici*	Clearing away heat and detoxifying, relieving swelling, dredging the collaterals to stop pain	Enhancing the immunity of inflammatory mice, reducing the IL-10 level to be normal, increasing the phagocytosis index and phagocytosis rate of macrophages, adjusting the balance of microflora, and thus enhancing the immune function
The combined appication of *Guizhi Fuling* pills and *Wuwei Xiaodu* Decoction [22]	*Ramulus Cinnamomi*, *Sclerotium Poriae Cocos*, *Radix Paeoniae Rubra*, *Cortex moutan Radicis*, *Semen Persicae*, *Flos Lonicerae*, *Flos Dendranthematis Indici*, *Herba Violae Philippicae*, *Radix Semiaquilegiae*, *Fructus Forsythiae Suspensae*, *Herba Leonuri Japonici*, *Rhizoma Alismatis*	Clearing away heat and detoxifying, activating blood and dredging the collaterals to stop pain	Reducing SUA and CRP levels, shortening the treatment time and reducing clinical side effects
*Zhuye Shigao* Decoction [23]	*Folium Phyllostachydis Henonis*, *Gypsum Fibrosum*, *Rhizoma Pinelliae Praeparatum*, *Radix Ginseng*, *Radix Ophiopogonis*, *Oryza Sativa* L., *Radix et Rhizoma Glycyrrhizae*	Clearing away heat and nourishing Yin, invigorating the spleen and excreting dampness	Improving erythrocyte sedimentation rate, C-reactive protein levels, and reducing the incidence of adverse reactions
The modified *Zhuye Shigao* Decoction [54]	*Folium Phyllostachydis Henonis*, *Gypsum Fibrosum*, *Rhizoma Pinelliae Praeparatum*, *Radix Adenophoreae Strictae*, *Radix Ophiopogonis*, *Rhizoma Anemarrhenae*, *Caulis Piperis Kadsura*e, *Cortex Erythrinae Variegatae*, *Caulis Mahoniae Japonicae*, *Herba Epimedii Brevicomus*, *Semen Coicis*, *Radix Angelicae Biserratae*, *Radix Achyranthis Bidentatae*, *Radix et Rhizoma Glycyrrhizae*	Clearing away heat and draining dampness, nourishing yin and dredging the collaterals to stop pain	Down-regulating the serum levels of IL-1β and Caspase-1 and the expression levels of Pro-IL-1 and Pro-caspase-1, inhibiting the inflammation induced by sodium urate crystal.
*Qingre Chubi* Decoction A [24, 25]	*Herba Aristolochiae Mollissimae*, *Caulis Sinomenii*, *Radix Cudraniae Cochinchinensi*s, *Rhizoma* Atractylodes *Lanceae*, *Rhizoma Anemarrhenae*	Dispelling wind and draining dampness, and dredging the collaterals to stop pain	Reducing leukocytes, ESR, CRP, inhibiting NF-κB expression, releasing IL-1β, TNF-α and inhibiting monocyte activity
*Qingre Chubi* Decoction B [26, 27]	*Caulis Sinomenii*, *Caulis Piperis Kadsura Ramulus Mori*, *Radix Stephaniae Tetrandrae*, *Herba Lycopi Hirti*, *Radix et Rhizoma Clematidis Chinensis*, *Rhizoma Smilacis Glabrae*, *Lumbricus*	Dispelling wind and draining dampness, and dredging the collaterals to stop pain	
*Tongfengshu* capsule [28]	*Radix et Rhizoma Rhei Palmati*, *Herba Plantagini*, *Rhizoma Alismatis*, *Radix Cyathulae*, *Radix Stephaniae Tetrandrae*	Promoting blood circulation and dredging the collaterals to stop pain, inducing diuresis and excreting dampness, and relieving swelling and pain	Reducing uric acid level; inhibiting HMGB1 and RAGE levels, reducing the level of PG in local inflammatory tissue by inhibiting the synthesis or release of PGE_2_, reducing the production of inflammatory cytokine TNF-α and anti-lipid oxidation, decreasing the levels of IL-1β and TNF-α, inhibiting the expression of MDA and NO, and promoting the release of SOD
The modified *Guizhi Shaoyao Zhimu* Decoction [30]	*Rhizoma Smilacis Glabrae*, *Radix Achyranthis Bidentatae*, *Rhizoma Dioscoreae*, *Rhizoma Zingiberis Recens*, *Rhizoma Atratylodis Macrocephalae*, *Radix* Ssaposhnikoviae, *Ramulus Cinnamomi*, *Radix Aconiti Praeparata*, *Rhizoma Anemarrhenae*, *Radix Ranunculi Polii*, *Ramulus Mori*, *Herba Ephedrae Sinicae*, *Radix et Rhizoma Glycyrrhizae*	Clearing away heat and nourishing yin, dispelling wind and cold, draining dampness and dredging the collaterals to stop pain	Reducing the levels of leukocyte, CRP, IL-6, IL-8, ESR, and SUA, and inhibiting inflammation and uric acid metabolism
*Guizhi Shaoyao Zhimu* Decoction [70]	*Ramulus Cinnamomi*, *Radix Ranunculi Polii*, *Radix et Rhizoma Glycyrrhizae*, *Herba Ephedrae Sinicae*, *Rhizoma Zingiberis Recens*, *Rhizoma Atratylodis Macrocephalae*, *Rhizoma Anemarrhenae*, *Radix Saposhnikoviae*, *Radix Aconiti Praeparata*	Dredging the collaterals to stop pain, dispelling cold and draining dampness, and clearing away heat and nourishing yin	Reducing the expression of TLR-2, TLR-4, NLRP3 receptor, My D88 and ASC protein, increasing the expression of PPAR-γ and IκB-α, inhibiting the differentiation and maturation of IL-1β and the activation of NF-κB, and reducing the expression of inflammatory factors like My D88 and NLRP3
*Tongyang Mizhuo* Decoction [34]	*Radix Ginseng*, *Rhizoma Zingiberis*, *Ramulus Cinnamomi*, *Rhizoma Atratylodis Macrocephalae*, *Sclerotium Polypori Umbellati*, *Sclerotium Poriae Cocos*, *Rhizoma Alismatis*, *Rhizoma Dioscoreae*, *Rhizoma Smilacis Glabrae*, *Radix et Rhizoma Clematidis Chinensis*, *Radix Achyranthis Bidentatae*	Warming yang and discharging dampness	
*Tongfengan* mixture [32]	*Herba Lysimachiae*, *Semen Sinapis*, *Thallus Ulvae Fasciatae*, *Spora Lygodii*, *Rhizoma Smilacis Glabrae*, *Flos Carthami*, *Radix Paeoniae Rubra*, *Pseudobullbus Cremastrae*, *Rhizoma Alismatis*, *Rhizoma Corydalis*	Softening hard lumps and dispel nodes, clearing heat and draining dampness, promoting blood circulation and dredging the collaterals to stop pain	Relieving the swelling of the ankle joint of rats, reducing the number of MSU crystals in the ankle joint of rats, inhibiting the secretion of inflammatory factors like IL-1β, TNF-α and NF-κB in the synovial tissue of the ankle joint of rats by clearing urate crystals, and reducing the inflammation in gout arthritis
*Quzhuo Tongbi* Decoction A [72]	*Rhizoma Smilacis Glabrae*, *Rhizoma Dioscoreae*, *Stigma Maydis*, *Semen Coicis*, *Rhizoma Curcumae Longae*, *Herba Taxilli Chinensis*, *Rhizoma Corydalis*, *Folium Pyrrosiae*, *Herba Plantaginis*	Invigorating spleen and kidney, promoting blood circulation and dredging the collaterals to stop pain, and draining dampness	Regulating some intestinal bacteria with abnormal uric acid metabolism, such as *Staphylococcus* which encodes uricase, reduces uric acid level, serum Cr and serum XOD levels
*Quzhuo Tongbi* Decoction B [74]	*Radix Aconiti*, *Rhizoma Smilacis Glabrae*, *Radix Bupleuri Chinensi*s, *Rhizoma Atratylodis Macrocephalae*, *Radix Astragali*, *Caulis Lonicerae*, *Radix Paeoniae Rubra*	Invigorate the spleen and draining dampness, clearing away heat and detoxifying, dredging the collaterals to stop pain	Blocking the sensation of peripheral nerve to pain and inhibiting the production of PGE_2_ in inflammatory mediators
*Peiyuan Huazhuo* formula [35]	*Rhizoma Polygonatum Sibirici*, *Semen Cuscutae*, *Radix Astragali Mongolici*, *Rhizoma Smilacis Glabrae*, *Semen Coicis*, *Rhizoma Corydalis*	Strengthening healthy qi and inducing diuresis	Relieving lipid metabolism disorders, inhibiting inflammatory reactions, preventing gout attacks, and preventing renal damage in patients with gout

## Gout and *Bi* syndrome patterns

In TCM, gout pertains the category of *Bi* pattern [13]. In general, the manifestations of *Bi* pattern are mainly characterized as pain, numbness and heaviness of muscles, tendons and joints or swelling of joints with hot sensation and limitation of movement [14]. According to TCM, the primary causes are the deficiency of healthy qi and the invasion of pathogenic factors like wind, cold, dampness or heat [12]. It is recorded in *Treatise on Blood Trouble* that the transportation and transformation function of spleen is closely related to the disease mechanism of gout. The deficiency of spleen qi could cause obstruction in the digestion system and lead to the buildup of waste products like uric acid in the blood. In addition, the evidences from clinical trials suggested that the dampness accumulation and blood stasis in the meridian would result in the swelling and pain of joint and the buildup of uric acid in the blood [13]. Because of the individual difference of body constitution and different pathogenic factors, there are various types of *Bi* pattern. For example, people with yang-deficiency constitution are usually affected by the combined invasion of wind, cold and dampness, and therefore there would be a *Bi* pattern of cold-dampness type, while people with yang-predominance constitution or yin-deficiency and yang-hyperactivity constitution are usually affected by the combined invasion of wind, dampness and heat, and finally there would be *Bi* pattern of damp-heat type. Among those factors, dampness and heat are the most common external causes of acute gout, while the deficiency of healthy qi and the invasion of dampness are the most common external causes for gout during remission.

## The clinical application of TCM formula in the treatment of gout

**Fig. 2:**
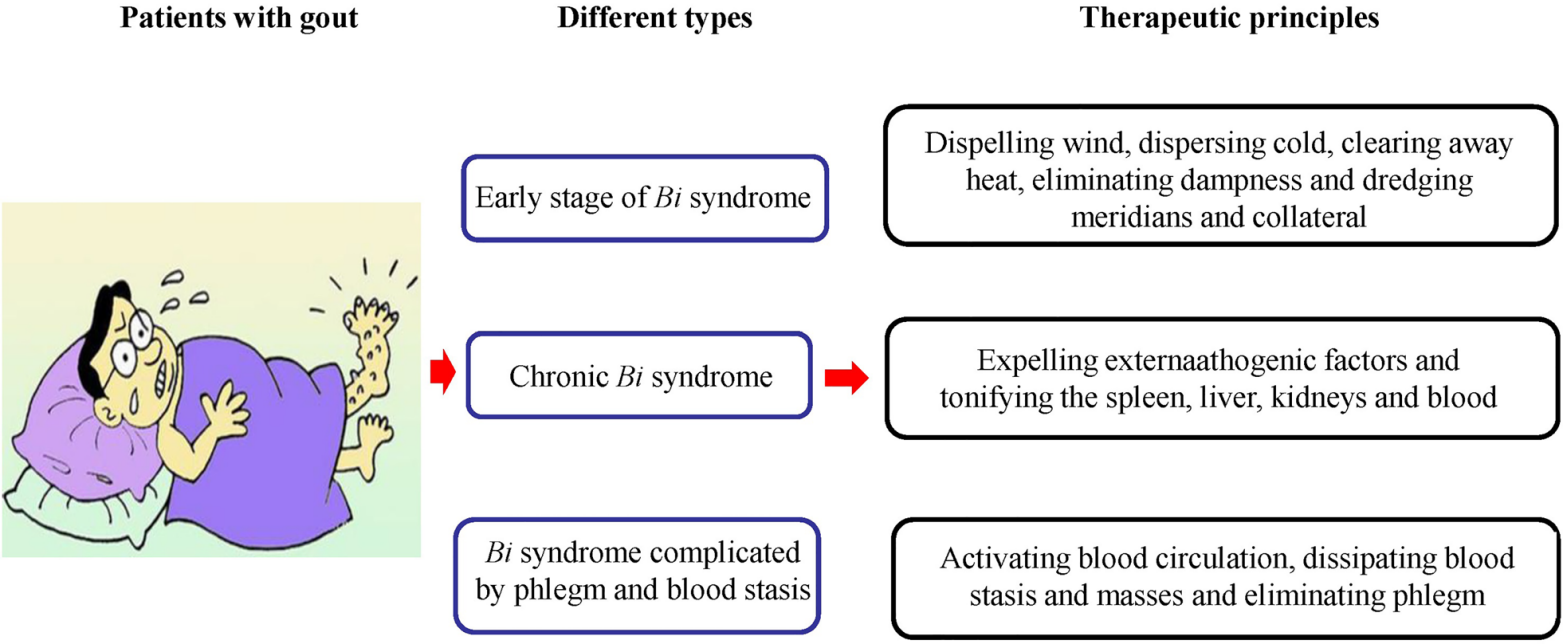
The main different types of and the therapeutic principles of gout. There are three different types of gout based on syndrome differentiation

In the early stage of *Bi* pattern, the therapeutic principles are expelling pathogenic factors, for example, dispelling wind, dispersing cold, clearing away heat, eliminating dampness and dredging meridians and collaterals [15]. For the patient suffering from *Bi* pattern for long time or the patient with weak body constitution, in addition to the therapy for expelling pathogenic factors, tonifying the spleen, liver, kidneys and nourishing blood should also be applied. For patients with gout complicated by phlegm and blood stasis, activating blood circulation, dissipating blood stasis and masses and eliminating phlegm should also be applied (Fig. 2) [16].

### The clinical application of TCM formula in treating acute gout

In recent years, TCM formulas are often modified or combined with chemical drugs to treat gout with fewer side effects, and have been regarded as the promising treatments [17]. Among these, *Simiao* Powder as well as the modified ones like *Jiawei Simiao* Powder, *Tongfeng* Decoction, *Danxi Tongfeng* Decoction are widely used for gout of damp-heat type. Chen et al. [17] found that the TCM formulas combined with chemical drugs showed better effect than chemical drugs applied alone. Similarly, the research from Huang et al. [18] suggested that the improvement of symptom score in the group treated with oral administration of *Jiawei Simiao* Powder and external application of *Jinhuang* Cream was more significant than that in the group treated with chemical drugs alone. In the group treated with TCM formula, the effective rate was higher than that in the group treated with chemical drugs, and the adverse reaction rate was significantly lower than that in the group treated with chemical drugs. Based on *Simiao* Powder, *Tongfeng* Decoction is invented and widely used. Wu et al. [19] found that after the administration of *Tongfeng* Decoction and celecoxib capsule, the acute inflammatory indicators in the blood sample of the gout patients were reduced, the inflammatory response was inhibited, the uric acid content was reduced, and the side effects were also reduced. In another clinical study, it was also found that the *Danxi Tongfeng* Decoction (the modified *Simiao* Powder) was better than celecoxib capsules in reducing the erythrocyte sedimentation rate (ESR) and serum uric acid (SUA) and prolonging the pain relieving time [20]. The above three TCM formulas with the effect of clearing away heat and draining dampness, and dredging the collaterals to stop pain, are modified from *Simiao* Powder.

*Wuwei Xiaodu* Decoction is an effective formula with the effect of clearing heat and detoxifying for treating patients with joint pain and obvious swelling. Yu et al. [21] applied modified *Wuwei Xiaodu* Decoction combined with colchicine tablets to treat the gout arthritis of damp-heat type, and found that the effective rate (86.00%) was higher than that in the control group (66.67%). Meanwhile, the combined application could effectively relieve acute joint pain, reduce swelling, relieve pain and improve joint function. Besides, the combined application of *Guizhi Fuling* Pill and *Wuwei Xiaodu* Decoction showed better effect than the combined application of meloxicam tablets and sodium bicarbonate tablets [22]. They have found that the effective rate was 97.60% in the group treated with TCM formulas, which was higher than that in the group treated with chemical drugs, and in the group treated with TCM formulas the pain degree was significantly reduced, the swelling score, SUA and C-reactive protein (CRP) levels were lower than those in the group treated with chemical drugs.

Patients with acute attack of gout usually show severe joint pain, high skin temperature, thirsty sensation, white or yellow coating on the tongue, and there are various therapeutic methods or formulas in TCM. Among them, *Zhuye Shigao* Decoction with the function of clearing away heat and nourishing yin, invigorating the spleen and draining dampness, is of good efficacy on these patients. In a clinical study by Li et al. [23], *Zhuye Shigao* Decoction combined with colchicine tablets and celecoxib capsules showed better effect than celecoxib capsules, the effective rate was increased from 62.86% to 91.43%, and the gastrointestinal reactions such as nausea, vomiting and diarrhea were reduced.

In another clinical reports, it is also illustrated that combined application of *Qingre Chubi* Decoction and etoricoxib tablets was better than etoricoxib tablets used alone to treat acute gout [24, 25]. *Qingre Chubi* Decoction, with the function of dispelling wind and draining dampness, and dredging the collaterals to stop pain, has good effect for patients with gout affected by the combined invasion of wind, dampness and heat [24, 25]. There are two formulas named as *Qingre Chubi* Decoction which are of different compositions and similar effects. One *Qingre Chubi* Decoction composed of *Herba Aristolochiae Mollissimae*, *Caulis Sinomenii*, *Radix Cudraniae Cochinchinensi*, *Rhizoma Atractylodis Lanceae* and *Rhizoma Anemarrhenae*, could relieve the clinical symptoms and signs effectively, and improve inflammation indexes more obviously than the chemical drugs. Another *Qingre Chubi* Decoction, composed of *Caulis Sinomenii*, *Caulis Piperis Kadsura*, *Ramulus Mori*, *Radix Stephaniae Tetrandrae*, *Herba Lycopi Hirti*, *Radix et Rhizoma Clematidis*, *Rhizoma Smilacis Glabrae* and *Lumbricus*, has a good therapeutic effect for gout and low incidence of adverse reactions [26, 27].

Wen et al. [28] proposed that the increased level of SUA was due to water-dampness and phlegm-dampness blocking the meridians and collaterals, and dysfunction of qi and blood from TCM perspective. He found that *Tongfengshu* Capsule could promote blood circulation and urination to reduce SUA with a total effective rate of 90.59%. Tang et al. [29] found that in patients treated with *Tongfengshu* Capsule combined with chemical drugs, symptoms and signs were relieved more obviously than in patients treated with chemical drugs alone, and the recurrence rate was lower than that in patients treated with chemical drugs 4 or 8 weeks after the treatment.

In the *Synopsis of Golden Chamber* the therapeutic principles for gout were dispelling cold and draining dampness, and clearing away heat and nourishing yin. *Guizhi Shaoyao Zhimu* Decoction is applied in clinic guided by those principle. Zhu et al. [30] found that the pain relieving time, number of relapses within 6 months and the TCM syndrome score in patients treated with the combined application of *Guizhi Shaoyao Zhimu* Decoction, celecoxib capsules and colchicine tablets, were lower than those treated with chemical drugs alone. He et al. [31] compared the modified *Guizhi Shaoyao Zhimu* Decoction with celecoxib capsules, and found that the pain and swelling were obviously relieved, and the activity restriction scores were significantly improved. Liu et al. [32] used *Tongfengan* Mixture according to the method of softening hard lumps and dispelling nodes to treat acute gout and found that this combination could reduce SUC, the severity and frequency of attacks. In conclusion, TCM formulas has better clinical efficacy and higher safety than chemical drugs in the treatment of gout.

### The clinical application of TCM formulas in the treatment of gout during the remission stage

During the remission stage of gout, the disease mechanism of TCM usually involves spleen deficiency with dampness accumulation. From TCM perspective, the abnormal production and excretion of uric acid is related to the dysfunctional of spleen, and tends to result in the increased content of SUA [33]. Qin et al. [34] used *Tongyang Mizhuo* Decoction, composed of *Lizhong* Pills and *Wuling* Powder to treat gout during the remission stage and found that the total effective rate was 94.4% and the SUA level was significantly reduced after the treatment. Emerging evidences from clinical observations indicated that patients during the remission stage were characterized by spleen deficiency with dampness accumulation. *Quzhuo Tongbi* Decoction is often used to treat spleen deficiency with dampness accumulation pattern and therefore it is suitable to treat gout during remission stage [33].

According to the TCM theory of “treating disease before its onset”, *Peiyuan Huazhuo* Decoction, is mainly used to prevent renal damage in patients with gout. TCM doctors found that the symptoms and the renal damage were relieved after the combination therapy, suggesting that the application of *Peiyuan Huazhuo* Decoction had the function of strengthening healthy qi and inducing diuresis [35].

In conclusion, TCM formulas commonality is promoting the body resistance and eliminating pathogenic factors. Different TCM formulas are applied to achieve the relative equilibrium of yin-yang based on the heterogeneity of body constitution and pathogenic factors.

## The pharmacological mechanism of TCM formulas in the treatment of gout

Emerging evidences have suggested that gout caused by monosodium urate deposition is related to disorders of purine metabolism and reduced uric acid excretion, so it is classified as a metabolic rheumatism. However, in recent studies it has been found that the pathogenesis is closely related to the mechanism of “metabolism-inflammation-immunity” [36]. TCM treatment for gout, especially the formulas from *Synopsis of Golden Chamber* could interfere the mechanism of “metabolism-inflammation-immunity” [37].

### The pharmacological mechanism of Simiao Powder and it’s modification in the treatment of gout

*Simiao* Powder is composed of *Cortex Phellodendri Amurensis*, *Semen Coicis*, *Radix Achyranthis Bidentatae and Rhizoma Atractylodis Lanceae.* All the herbs in the *Simiao* Powder are also the major ingredients in the *Jiawei Simiao* Powder, *Tongfeng* Decoction *and Danxi Tongfeng* Decoction. According to TCM theory, *Cortex Phellodendri Amurensis* is bitter in flavor and has curative effects on the kidneys, bladder and large intestine. The main medicinal components of *Cortex Phellodendri Amurensis* are alkaloids such as berberine, lipoid and sterol [38], with the function of anti-inflammation, bacteriostasis, cellular immune regulation, anti-gout and inhibiting the apoptosis of articular chondrocytes [39]. *Rhizoma Atractylodis Lanceae* is pungent and bitter in flavor, and usually works on the kidneys, bladder and large intestine. It has the function of anti-bacterium, anti-inflammation, reducing blood glucose level and suppressing central nervous system [40]. Satoh et al. [41] found that *Rhizoma Atractylodis Lanceae* could inhibit the activity of sodium–potassium-ATPase, prevent the absorption of water and sodium ions, and thus to promote urination and excretion.

*Radix Achyranthis Bidentatae* is sweet in flavor, has the function of reinforcing liver and kidneys, strengthening muscles and bones, and promoting urination, and usually works on the liver and kidneys. It is reported that *Radix Achyranthis Bidentatae* could promote the excretion of uric acid, improve the cellular and humeral immunity as well as the non-specific immune function [42].

The combination of *Cortex Phellodendri Amurensis*, *Radix Achyranthis Bidentatae* and *Rhizoma Atractylodis Lanceae* can reduce the levels of tumor necrosis factor (TNF)-α, interleukin (IL)-6 and IL-8 in synovial tissue. Having the function of anti-inflammatory and inhibiting cyclooxygenase-2 (COX-2), *Semen Coicis Alba*is could works on regulating purine metabolism and reducing uric acid formation [43]. After *Semen Coicis Alba*is is added, this combination of four herbs becomes *Simiao* Powder, which has good effect on the patient with gout of dampness-heat type [44]. Wang et al. [45] suggested that modified *Sanmiao* Powder could inhibit the content of inflammatory factors in the joint fluid of rats with acute gout arthritis.

Although *Jiawei Simiao* Powder, *Tongfeng* Decoction and *Danxi Tongfeng* Decoction are all invented based on *Simiao* Powder, the pharmacological mechanisms of these TCM formulas are different. Oral administration of *Jiawei Simiao* Powder and combined with external application of *Jinhuang* Cream could relieve the inflammation by reducing the levels of ESR, CRP and IL-8 [18]. *Tongfeng* Decoction could effectively reduce SUA, inhibit inflammatory response, halt the progression of the disease, and have the function of reducing uric acid [19]. *Danxi Tongfeng* Decoction could reduce ESR and SUA levels, and has a good analgesic effect [20].

### The pharmacological mechanism of Wuwei Xiaodu Decoction in the treatment of gout

*Wuwei Xiaodu* Decoction, composed of *Flos Lonicerae*, *Flos Dendranthematis Indici*, *Herba Taraxaci Mongolici*, *Herba Violae Philippicae* and *Nudicaulous Grounsel Herb*, is firstly recorded in *Golden Mirror of the Medical Tradition*. It is recorded that *Flos Lonicerae*, *Flos Dendranthematis Indici* and *Nudicaulous Grounsel Herb* have the function of anti-inflammation and immunomodulation [46–48]. Some researchers find that *Herba Taraxaci Mongolici* has the function of anti-bacteria, anti-oxidation, anti-inflammation, diuresis, anti-allergy, promoting immunity, and is beneficial for the stomach [49]. Because of its strong effect of anti-pathogenic microorganisms, *Herba Violae Philippicae* is often used in the treatment of ulcers and carbuncle with symptoms like swelling and pain [50]. It is believed that *Wuwei Xiaodu* Decoction can enhance the immunity of diseased mice and reduce the IL-10 to the normal level [51]. Besides, another report also illustrates that *Wuwei Xiaodu* Decoction could increase the phagocyte index and phagocyte rate of macrophages, adjust the balance of flora and thus enhance the immune function [52].

### The pharmacological mechanism of Zhuye Shigao Decoction in the treatment of gout

*Zhuye Shigao* Decoction is composed of *Folium Phyllostachydis Henonis*, *Gypsum Fibrosum*, *Rhizoma Pinelliae Praeparatum*, *Radix Ginseng*, *Radix Ophiopogonis*, *Oryza Sativa* L., *Radix Ophiopogonis Japonicir* and *Radix et Rhizoma Glycyrrhizae.* Wang et al. [53] held that gout was caused by dampness accumulation and heat blocking the meridians and collaterals, so he adopted the methods of clearing heat and resolving dampness, unblocking the collaterals and relieving pain, and applied *Zhuye Shigao* Decoction for the treatment. Chen et al. [54] found that after being treated with modified *Zhuye Shigao* Decoction, serum levels of IL-1β and caspase-1 were reduced, and the expression levels of pro-IL-1 and pro-caspase-1 were down-regulated, suggesting that the modified *Zhuye Shigao* Decoction could inhibit the inflammation induced by sodium urate crystals, which may be related to the intervention of IL-1 signaling pathway.

### The pharmacological mechanism of Qingre Chubi Decoction in the treatment of gout

There are two formulas named as *Qingre Chubi* Decoction which are of different compositions and similar effects. In one *Qingre Chubi* Decoction, the ingredients include *Herba Aristolochiae Mollissimae*, *Caulis Sinomenii*, *Radix Cudraniae Cochinchinensi*, *Rhizoma Atractylodis Lanceae*, and *Rhizoma Anemarrhenae*. Another *Qingre Chubi* Decoction is composed of *Caulis Sinomenii*, *Caulis Piperis Kadsura*, *Ramulus Mori*, *Radix Stephaniae Tetrandrae Herba Lycopi Hirtiaa*, *Radix et Rhizoma Clematidis*, *Rhizoma Smilacis Glabrae* and *Lumbricus.* Wang et al. [55] confirmed that the first *Qingre Chubi* Decoction could relieve the symptoms of patients such as feverish sensation, joint swelling and pain, and reduce the inflammatory indicators such as ESR and CRP. Some studies suggested that *Qingre Chubi* Decoction might inhibit the release of IL-1β and TNF-α to ameliorate the acute inflammatory response of gout [56]. Additionally, *Qingre Chubi* Decoction inhibited the proliferation of THP-1 cells stimulated by MSU crystals. The inhibition of monocyte activity and IL-1β may be one of the anti-inflammatory mechanisms of *Qingre Chubi* Decoction [57].

### The pharmacological mechanism of Tongfengshu Capsule in the treatment of gout

*Tongfengshu* Capsule, composed of *Radix et Rhizoma Rhei Palmati*, *Semen Plantaginis*, *Rhizoma Alismatis*, *Radix Achyranthis Bidentatae* and *Radix Stephaniae Tetrandrae*, is a Chinese patent medicine. Rheum emodin, one chemical component from *Radix et Rhizoma Rhei Palmati*, could inhibit the expression of NOD-like receptor family pyrin domain-containing 3 (NLRP3) and IL-1 mRNAs [58] and inhibit the activation of extra cellular signal–regulated kinases 1 and 2 (ERK1/2) and p38 nitrogen-activated protein kinases (p38 MAPK) signaling pathways by inhibiting the expression of ERK1/2 nucleus and p38MAPK, thereby reducing the release of inflammatory factors [59]. *Semen Plantaginis* and *Rhizoma Alismatis* could promote urination, and reduce swelling and pain. Among them, *Semen Plantaginis*, rich in mullein glycolic anhydride, could promote uric acid secretion, reduce the creatinine level, scavenge reactive oxygen species (ROS) and improve renal function [60]. In addition, it is reported that the ethanol extract of *Rhizoma Alismatis* could reduce SUA level without damaging renal function [61]. *Radix Stephaniae Tetrandrae* has the function of dispelling wind, relieving pain and inducing diuresis to alleviate edema. *Tetrandrine*, the active ingredient of *Radix Stephaniae Tetrandrae* could inhibit the inflammatory response of cells, and this anti-inflammation mechanism may be related to inhibiting nuclear factors such as NF-κB, reducing IL-6 and TNF-α levels, and promoting the expression of anti-inflammatory cytokines such as IL-10 [62]. It is also reported that *Radix Achyranthis Bidentatae* could increase superoxide dismutase (SOD) activity, reduce serum lipid peroxide level, and regulate immunity and metabolism of the body [63]. Based on the previous study, the anti-inflammation mechanism of *Tongfengshu* Capsule may be related to inhibiting the synthesis or release of prostaglandin E_2_ (PGE_2_), reducing the production of inflammatory cytokines like TNF-α and resisting lipid peroxidation [64]. It was also found that *Tongfengshu* capsule could significantly relieve symptoms of joints in rats with acute gout arthritis, suggesting the mechanism might be related to reducing IL-1β and TNF-α levels, inhibiting the expression of malonic dialdehyde (MDA) and nitrogen oxide (NO), and promoting the release of SOD [65]. Besides, some researchers proposed that the therapeutic mechanism of *Tongfengshu* Capsule in the treatment of gout arthritis was related to the down-regulation of serum levels of high mobility group protein 1 (HMGB l) and receptor for advanced glycosylation end-products (RAGE) in patients with gout [65].

### The pharmacological mechanism of Guizhi Shaoyao Zhimu Decoction in the treatment of gout

*Guizhi Shaoyao Zhimu* Decoction, firstly recorded in the TCM classics, *Synopsis of the Golden Chamber*, is composed of *Herba Ephedrae Sinicae*, *Ramulus Cinnamomi*, *Radix Saposhnikoviae*, *Largehead Atractylodes Rhizome*, *Radix Aconiti Lateralis Praeparata*, *Rhizoma Anemarrhenae*, *Radix Paeoniae Alba*, *Radix et Rhizoma Glycyrrhizae* and *Rhizoma Zingiberis Recens. Herba Ephedrae Sinicae*, could scavenge ROS and had significant antioxidant effects [65]. In addition to anti-bacterium, anti-virus and anti-inflammation effects [66, 67], *Ramulus Cinnamomi* and *Radix Saposhnikoviae* can also stimulate the secretion of saliva and gastric juice and stimulate sweat glands to dissipate heat [68]. *Largehead Atractylodes Rhizome* and *Radix Aconiti Lateralis Praeparata* have the function of warming channels to dispel coldness. It is recorded that *Rhizoma Anemarrhenae* and *Radix Paeoniae Alba* could nourish yin and clear heat. It took the researchers 5 years to find that *Guizhi Shaoyao Zhimu* Decoction has the function of relieving pain, anti-inflammation and healing bone injury. Besides, it also could induce apoptosis of synovial cell, regulate immunity, and promote uric acid excretion, and protect renal function to treat gout arthritis [69]. Some researchers proposed that the mechanism underlying the *Guizhi Shaoyao Zhimu* Decoction in the treatment of gout arthritis was related to reducing the expression of Toll-like receptor (TRL)-2, TRL-4, NLRP3 receptor, myeloid differentiation factor (MyD)88 and apoptosis-associated speck-like protein (ASC), increasing the expression of peroxisome proliferator-activated receptor (PPAR)-γ and inhibitor-κB (IκB)-α, inhibiting the differentiation and maturation of IL-1β and the activation of NF-κB [70].

### The pharmacological mechanism of Tongfengan Mixture in the treatment of gout

*Tongfengan* Mixture, composed of *Herba Lysimachiae*, *Semen Sinapis*, *Thallus Ulvae Fasciata*, *Spora Lygodii*, *Rhizoma Smilacis Glabrae*, *Flos Carthami*, *Radix Paeoniae Rubra*, *Pseudobullbus Cremastrae*, *Rhizoma Alismatis* and *Rhizoma Corydalis*, has the function of softening hardness and dissipating mass. It is found that in the rat models, *Tongfengan* Mixture can relieve the swelling, reduce the level of MSU crystals in the ankle joints, and reduce the inflammatory reaction, which may be achieved by reducing the inflammatory reaction in the blood [71]. Liu et al. [32] suggested that *Tongfeng* Mixture could inhibit the secretion of inflammatory factors like IL-1β, TNF-α and NF-κB in the synovial tissue of the ankle joint of rats by clearing away urate crystals, and reducing the inflammation.

### The pharmacological mechanism of Quzhuo Tongbi Decoction in the treatment of gout

There are two formulas name as *Quzhuo Tongbi* Decoction which are of different compositions and similar effects. In one *Quzhuo Tongbi* Decoction, the ingredients include *Rhizoma Smilacis Glabrae*, *Rhizoma Dioscoreae*, *Stigma Maydis*, *Semen Coicis*, *Rhizoma Curcumae Longae*, *Herba Taxilli Chinensis*, *Rhizoma Corydalis*, *Folium Pyrrosiae* and *Semen Plantaginis*. Another *Quzhuo Tongbi* Decoction is composed of *Radix Aconiti*, *Rhizoma Smilacis Glabrae*, *Radix Bupleuri Chinensi*, *Largehead Atractylodes Rhizome*, *Radix Astragali*, *Caulis Lonicerae* and *Radix Paeoniae Rubra*. It has been documented that some intestinal bacteria could cause abnormal uric acid metabolism [72]. Zhu et al. [73] found that the medium and high dosage of the first *Quzhuo Tongbi* Decoction can reduce the level of uric acid, serum creatinine and xanthine oxidase (XOD). Besides, it could also relieve inflammation symptoms, inhibit the production of PGE_2_, relieve pain sensation by blocking the sensation of peripheral nerves to pain stimuli [74].

### The pharmacological mechanism of Peiyuan Huazhuo Decoction in the treatment of gout

*Peiyuan Huazhuo* Decoction is composed of *Rhizoma Polygonatum Sibirici*, *Semen Cuscutae*, *Radix Astragali*, *Rhizoma Smilacis Glabrae*, *Semen Coicis* and *Rhizoma Corydalis.* Yang et al. [75] found that *Radix Astragali* could increase the glomerular filtration rate. It was also found that *Rhizoma Polygonatum Sibirici* not only had a good effect of lowering SUA levels, but also reduced blood lipid and glucose [35]. The herbs in this formula work together to treat lipid metabolism disorders, inhibit inflammatory reactions, prevent gout attack, and prevent renal damage in patients with gout.

## Conclusions and perspective

**Fig. 3:**
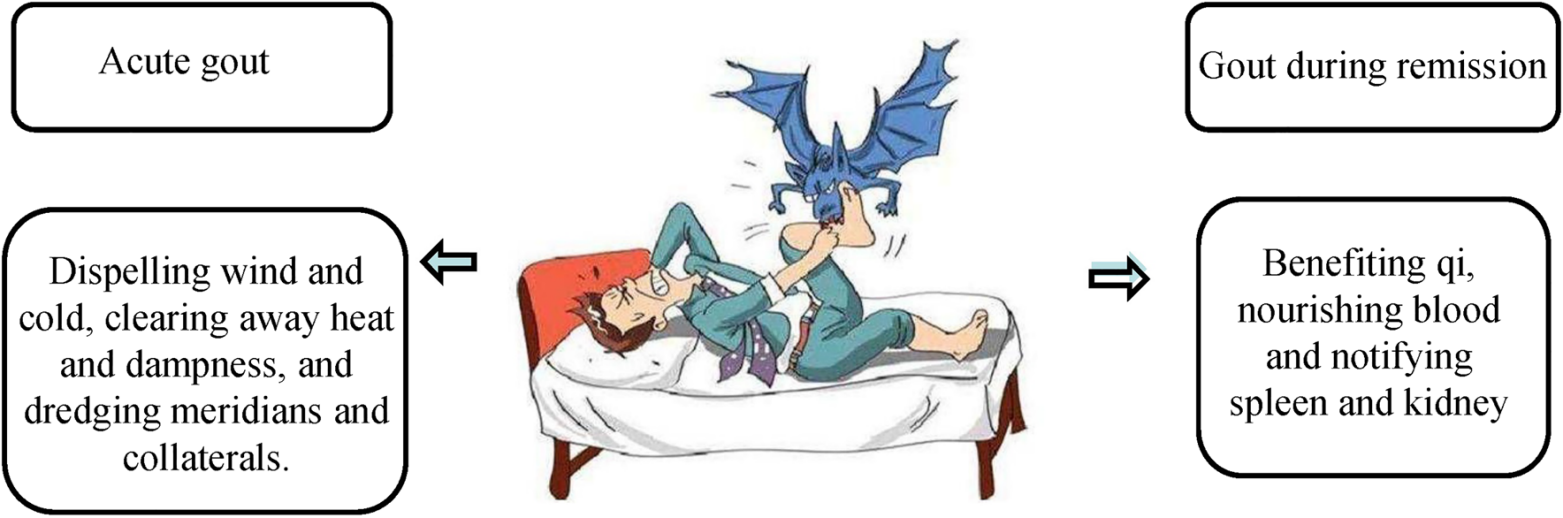
The main pharmacological mechanisms of the treatment of acute gout and gout during the remission. The disease of gout consists of the attack stage and remission stage, which should be treated differently based on disease mechanism of TCM

**Fig. 4:**
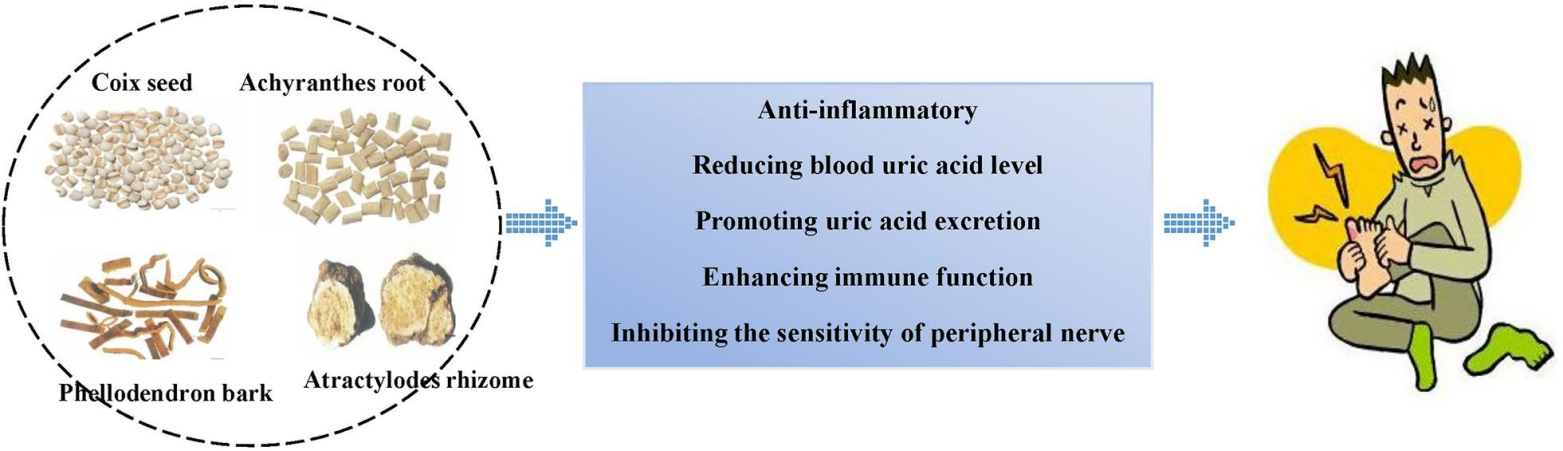
The main TCM formulas and the underlying pharmacological mechanisms of the treatment of gout. There are two ways to reduce the SUA level: one is to promote uric acid excretion, and the other is to reduce uric acid production

The etiology of gout is complex and there are various hypotheses about its pathogenesis. According to the disease mechanism of TCM, therapeutic principles like dispelling wind and cold, clearing away heat and dampness, and dredging meridians and collaterals are often applied to treat acute gout [16]. During the remission stage, benefiting qi, nourishing blood and tonifying spleen and kidney are often applied (Fig. 3) [44]. Inhibiting inflammatory factors in joint fluid, reducing blood uric and promotion of uric acid excretion, enhancing immune function and blocking the sensation of peripheral nerve are the main mechanism underly TCM treatment for gout (Fig. 4).

Compared with chemical drugs like colchicine, eloricoxib and celecoxib, TCM herbs have advantages in slowing down the severity and frequency of attacks, reducing the concentration of uric acid in blood with fewer side effects. Although TCM has a strong advantage in the treatment of gout, the application of TCM herbs or formulas should follow the principle of syndrome differentiation and treatment from TCM perspective.

In this review, we focus on the research data from animal and clinical studies in recent years, and summarize the TCM theory of gout, the clinical application, pharmacological mechanism and the chemical compounds of TCM formulas for gout treatment. By doing this we hope to help the researchers on exploring the mechanism of TCM treatment for gout and to develop agents more effectively for gout treatment.

## Data Availability

All the data used to support the findings of this study are available from the corresponding author upon reasonable request.

## Abbreviations

MSU: Monosodium urate
NSAIDs: Non-steroidal anti-inflammatory drugs
TCM: Traditional Chinese medicine
TNF-α: Tumor necrosis factor
IL-6: Interleukin
ESR: Erythrocyte sedimentation rate
CRP: C-reaction protein CRP
NLRP3: NOD-like receptor family pyrin domain-containing 3
MAPK: Mitogen-activated protein kinase
ROS: Reactive oxygen species
SOD: Superoxide dismutase
MDA: Malonic dialdehyde
NO: Nitrogen oxide
HMGB1: High mobility group protein 1
RAGE: Receptor for advanced glycosylation end-products
SUA: Serum uric acid
TRL: Toll-like receptor
MyD: Myeloid differentiation
ASC: Apoptosis-associated speck-like protein
PPAR: Peroxisome proliferator-activated receptor
PGE_2_: Prostaglandin E_2_
XOD: Xanthine oxidase
